# The Promyelocytic Leukemia Zinc Finger Transcription Factor Is Critical for Human Endometrial Stromal Cell Decidualization

**DOI:** 10.1371/journal.pgen.1005937

**Published:** 2016-04-01

**Authors:** Ramakrishna Kommagani, Maria M. Szwarc, Yasmin M. Vasquez, Mary C. Peavey, Erik C. Mazur, William E. Gibbons, Rainer B. Lanz, Francesco J. DeMayo, John P. Lydon

**Affiliations:** 1 Department of Molecular and Cellular Biology, Baylor College of Medicine, Houston, Texas, United States of America; 2 Department of Obstetrics and Gynecology, Baylor College of Medicine, Houston, Texas, United States of America; 3 Houston Fertility Specialists, Houston, Texas, United States of America; University of Missouri Columbia, UNITED STATES

## Abstract

Progesterone, *via* the progesterone receptor (PGR), is essential for endometrial stromal cell decidualization, a cellular transformation event in which stromal fibroblasts differentiate into decidual cells. Uterine decidualization supports embryo implantation and placentation as well as subsequent events, which together ensure a successful pregnancy. Accordingly, impaired decidualization results not only in implantation failure or early fetal miscarriage, but also may lead to potential adverse outcomes in all three pregnancy trimesters. Transcriptional reprogramming on a genome-wide scale underlies progesterone dependent decidualization of the human endometrial stromal cell (hESC). However, identification of the functionally essential signals encoded by these global transcriptional changes remains incomplete. Importantly, this knowledge-gap undercuts future efforts to improve diagnosis and treatment of implantation failure based on a dysfunctional endometrium. By integrating genome-wide datasets derived from decidualization of hESCs in culture, we reveal that the promyelocytic leukemia zinc finger (PLZF) transcription factor is rapidly induced by progesterone and that this induction is indispensable for progesterone-dependent decidualization. Chromatin immunoprecipitation followed by next generation sequencing (ChIP-Seq) identified at least ten progesterone response elements within the PLZF gene, indicating that PLZF may act as a direct target of PGR signaling. The spatiotemporal expression profile for PLZF in both the human and mouse endometrium offers further support for stromal PLZF as a mediator of the progesterone decidual signal. To identify functional targets of PLZF, integration of PLZF ChIP-Seq and RNA Pol II RNA-Seq datasets revealed that the early growth response 1 (EGR1) transcription factor is a PLZF target for which its level of expression must be reduced to enable progesterone dependent hESC decidualization. Apart from furnishing essential insights into the molecular mechanisms by which progesterone drives hESC decidualization, our findings provide a new conceptual framework that could lead to new avenues for diagnosis and/or treatment of adverse reproductive outcomes associated with a dysfunctional uterus.

## Author Summary

Following embryo attachment to the uterine epithelium, the underlying decidualized stroma is critical for further invasion by the conceptus into the maternal compartment. Because endometrial decidualization is required early in the continuum of events that lead to a successful pregnancy, abnormal decidualization can contribute not only to implantation failure or early miscarriage, but may initiate adverse reproductive outcomes that manifest in subsequent pregnancy trimesters. Genome-wide transcriptional changes by progesterone are known to underlie decidualization; however, the pivotal signals that functionally enable progesterone-driven decidualization are not fully known. Using an integrative analysis of genome-scale data along with studies on primary human endometrial stromal cells (hESCs), we reveal that the promyelocytic leukemia zinc finger (PLZF) transcription factor is rapidly induced by progesterone, and its induction is essential for progesterone-dependent hESC decidualization. Although PLZF in turn governs a remarkable array of target genes in the hESC, we demonstrate that PLZF tightly regulates the expression level of the early growth response 1 (EGR1) transcription factor, the perturbation of which compromises progesterone dependent decidualization. Together, our findings provide a new mechanistic perspective on progesterone action in the uterus which may furnish new opportunities for the formulation of more effective fertility solutions in the future.

## Introduction

For healthy couples, the chance for conception per menstrual cycle is only thirty-to-forty percent [1], underscoring the remarkable inefficiency of human reproduction. Moreover, approximately a third of pregnancies detected early by human chorionic gonadotropin assay fail to result in live births [2, 3], implicating early implantation failure as a causal factor for preclinical pregnancy loss. Implantation failure and early embryo miscarriage also undercut the full potential of assisted reproductive technologies (ARTs) which rely on the transfer of healthy embryos into a uterus that must in turn execute the requisite cellular and molecular changes to ensure the establishment of the fetomaternal interface [4]. Early functional impairment of the uterus is also implicated in recurrent pregnancy loss when parental chromosomal abnormalities, maternal thrombophilic disorders and anatomical uterine defects are first eliminated as causal factors [5]. Apart from contributing to early implantation failure, insufficient execution of normal uterine changes around the time of implantation is also thought to underpin adverse outcomes—pre-eclampsia and placental insufficiency, intrauterine fetal growth restriction, and preterm birth—in later trimesters of pregnancy, termed the “adverse ripple effect”; reviewed in [6].

Successful advancement of embryo implantation into a receptive endometrium requires decidualization of the endometrial stromal cell layer, a critical cellular transformation process in which quiescent stromal fibroblasts proliferate and differentiate into transient epithelioid decidual cells [7]. In addition to promoting local resistance to oxidative insults and gestational immunotolerance for the developing fetal allograft, decidual cells are thought to modulate the invasion of the embryonic trophoblast to a sufficient depth to establish fetoplacental circulation between the chorionic villi and the maternal intervillus space. Therefore, due to the indispensability of the decidualized stroma for placentation, inadequate endometrial decidualization is considered a critical yet poorly understood etiologic factor not only for early implantation failure but also for a broad spectrum of pregnancy disorders that manifest later.

Along with exposure to preovulatory and nidatory estradiol-17β (E2), the endometrial stroma requires progesterone (P4) “the hormone of pregnancy” and its nuclear receptor (the progesterone receptor (PGR)) for decidualization [6]. While many of the cellular events that underpin endometrial stromal cell decidualization are known, the key molecular signals that mediate P4-dependent decidualization of the human endometrium remain unclear. Integrating datasets from chromatin immunoprecipitation followed by next generation sequencing (ChIP-Seq) and RNA sequencing (RNA-Seq), we reveal that the promyelocytic leukemia zinc finger (PLZF) transcription factor is rapidly induced by P4 in human endometrial stromal cells (hESCs) and that this induction is indispensable for P4-driven hESC decidualization. Additionally, we show that PLZF tightly regulates the expression levels of the early growth response 1 (EGR1) transcription factor, the perturbation of which impairs hESC decidualization.

Together, our studies have uncovered a new mediator pathway of the P4 signal that enables quiescent hESCs to differentiate into decidual cells, a cellular transformation process that is essential for early pregnancy establishment.

## Results

### Progestin induction of PLZF expression during decidualization of human endometrial stromal cells

**Fig 1:**
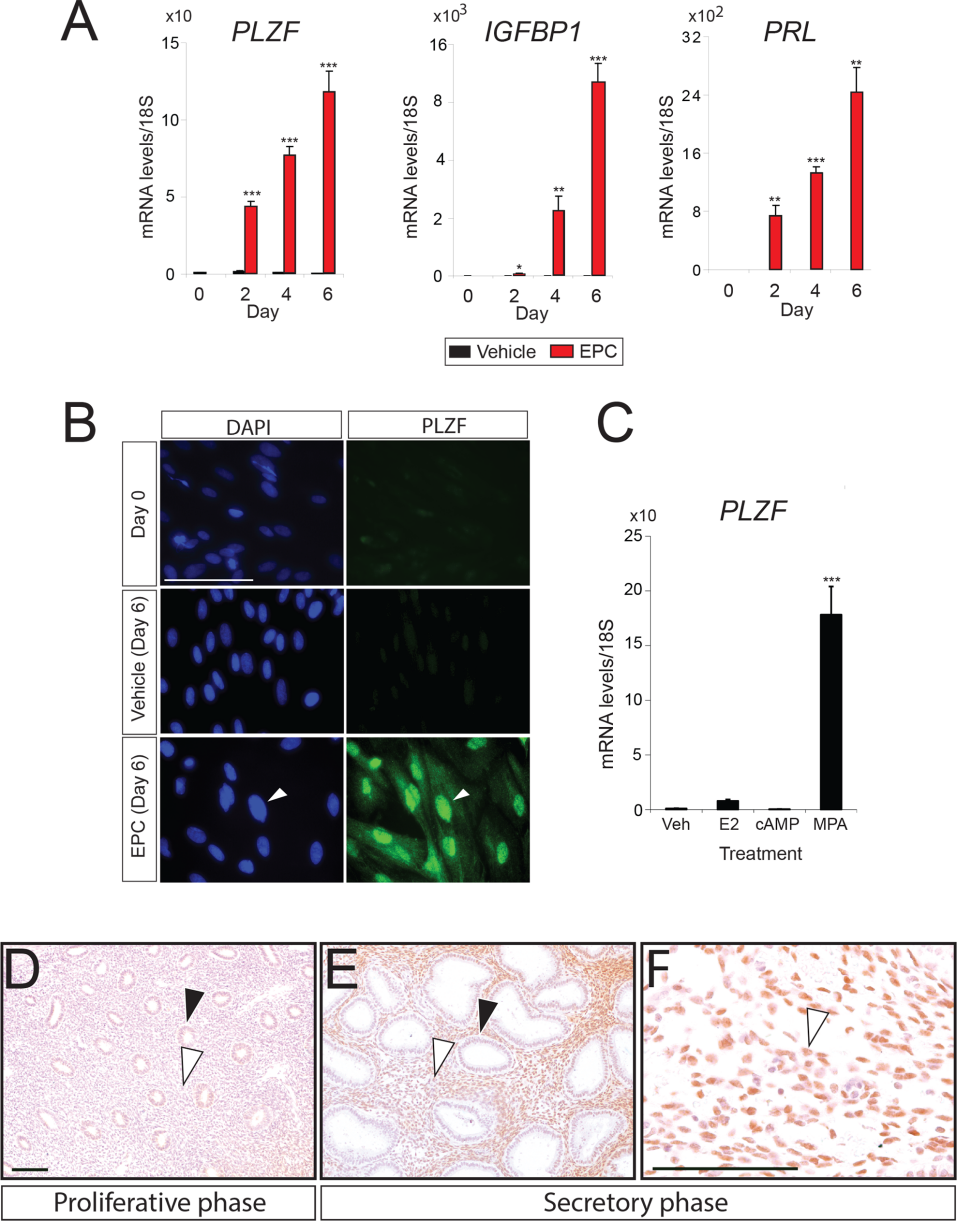
Progestin induction of PLZF expression in hESCs undergoing decidualization. **A)** Relative transcript levels of *PLZF*, *IGFBP-1*, *and PRL* in hESCs cultured with vehicle (black bar (control)) or the EPC cocktail (red bar) at the indicated time points of cell culture. Note: Day 0 is the first day that hESCs were treated with vehicle or the EPC cocktail. **B)** Immunofluorescent detection of PLZF protein expression in hESCs cultured with vehicle or the EPC cocktail at the indicated time points of culture. Left and right columns show images of the same cell field stained for DAPI and PLZF immunopositivity respectively. After 6 days of EPC treatment, note the prominent nuclear staining for PLZF expression in a decidualized hESC cell (arrowhead). **C)** Relative transcript levels of *PLZF* in hESCs cultured for 6 days in vehicle control, E2, cAMP, or MPA alone. Note: For each transcript type (panel A) or reagent (panel C), results are reported relative to day 0 or vehicle treatment group respectively as the mean ± standard error (SE) from triplicate samples from one subject (three subjects were tested in total). *P<0.05, **P<0.01, and ***P<0.001. **D-F)** Immunohistochemical analysis of PLZF expression in human endometrial tissue biopsied during proliferative phase (D) and secretory phase (E and F) of the menstrual cycle. Black and white arrowheads in panel D and E indicate glandular epithelial and stromal cells respectively; white arrowhead in panel F indicates the nuclear localization of PLZF protein in stromal cells. Scale bar in panel D denotes 100 μm and applies to panel E; scale bar in panel F represents 100 μm.

Comparing undecidualized hESCs with decidualized hESCs in culture, we recently revealed by RNA-seq analysis that *PLZF* (also known as *ZBTB 16*, *a* Kruppel C2H2-type zinc-finger transcription factor) is listed in the top 20 genes which are significantly induced in hESCs during decidualization [8] (**S1A Fig**). Additionally, we previously showed by microarray analysis that *Plzf* is present in the top 100 genes induced in total uterine tissue of the mouse in response to acute P4 administration [9], suggesting PLZF is a P4-responsive gene and important for decidualization. To address this proposal, an established cell culture-based model for decidualization of primary hESCs was used to determine the transcriptional induction profile of *PLZF* (**Fig 1A**). In response to a hormone decidual stimulus (17β-estradiol (E2), medroxyprogesterone acetate (MPA), and cAMP (EPC)), rapid induction of high transcript levels for *PLZF* was observed by quantitative real time PCR (**Fig 1A**). The induction of transcripts encoding insulin-like growth factor binding protein 1 (*IGFBP1*) and prolactin (*PRL*) (both established decidual biomarkers [10]) confirmed decidualization of hESCs by EPC at the molecular level. Consistent with PLZF’s role as a transcription factor, most of the induced PLZF protein expression was present in the nucleus of decidual cells following EPC treatment (**Fig 1B**). Similar to findings in **Fig 1A**, PLZF protein expression was not detected in pre-decidual hESCs or with vehicle treatment (**Fig 1B**). Treatment of hESCs with E2, cAMP, or MPA revealed that only MPA significantly induced *PLZF* transcription in hESCs (**Fig 1C**), providing strong support for the progestin in the decidual hormone cocktail as the key driver of *PLZF* induction in decidualizing hESCs. Moreover, PLZF transcript levels are induced with acute progestin treatment (as early as 2 hours post MPA administration), which is not dependent on new protein synthesis (**S1B and S1C Fig**). These results support PLZF as an early progestin responsive gene in hESCs. As further support for this conclusion, immunohistochemical analysis of human endometrial biopsy samples show that PLZF protein is highly expressed in the nucleus of the endometrial stromal cell during the P4-dominant secretory phase of the menstrual cycle but is expressed at low levels in endometrial tissue biopsied during the E2-dominant proliferative phase of the cycle (**Fig 1D–1F**). In the uterus of the ovariectomized mouse, *Plzf* transcript and protein levels are also significantly and rapidly induced by P4 (**S2 Fig**), validating our previous microarray analysis of this tissue which showed *Plzf* transcription is induced by short-term P4 exposure [9]. Transcript and protein levels for Plzf were detected as early as six hours following P4 administration (**S2A and S2B Fig**). Furthermore, expression levels of Plzf protein are low in the endometrium of the adult virgin mouse but are significantly increased in the uterine stromal compartment of the early pregnant mouse (**S2C Fig**). It should be noted that global knockout of *Plzf* in the mouse results in a number of embryonic and developmental phenotypes [11] that preclude its use for uterine studies. However, the above results support the proposal that PLZF acts as a progestin/P4 early response transcription factor in human and mouse uterine stromal cells.

### Progestin induction of PLZF in hESCs requires the progesterone receptor

**Fig 2:**
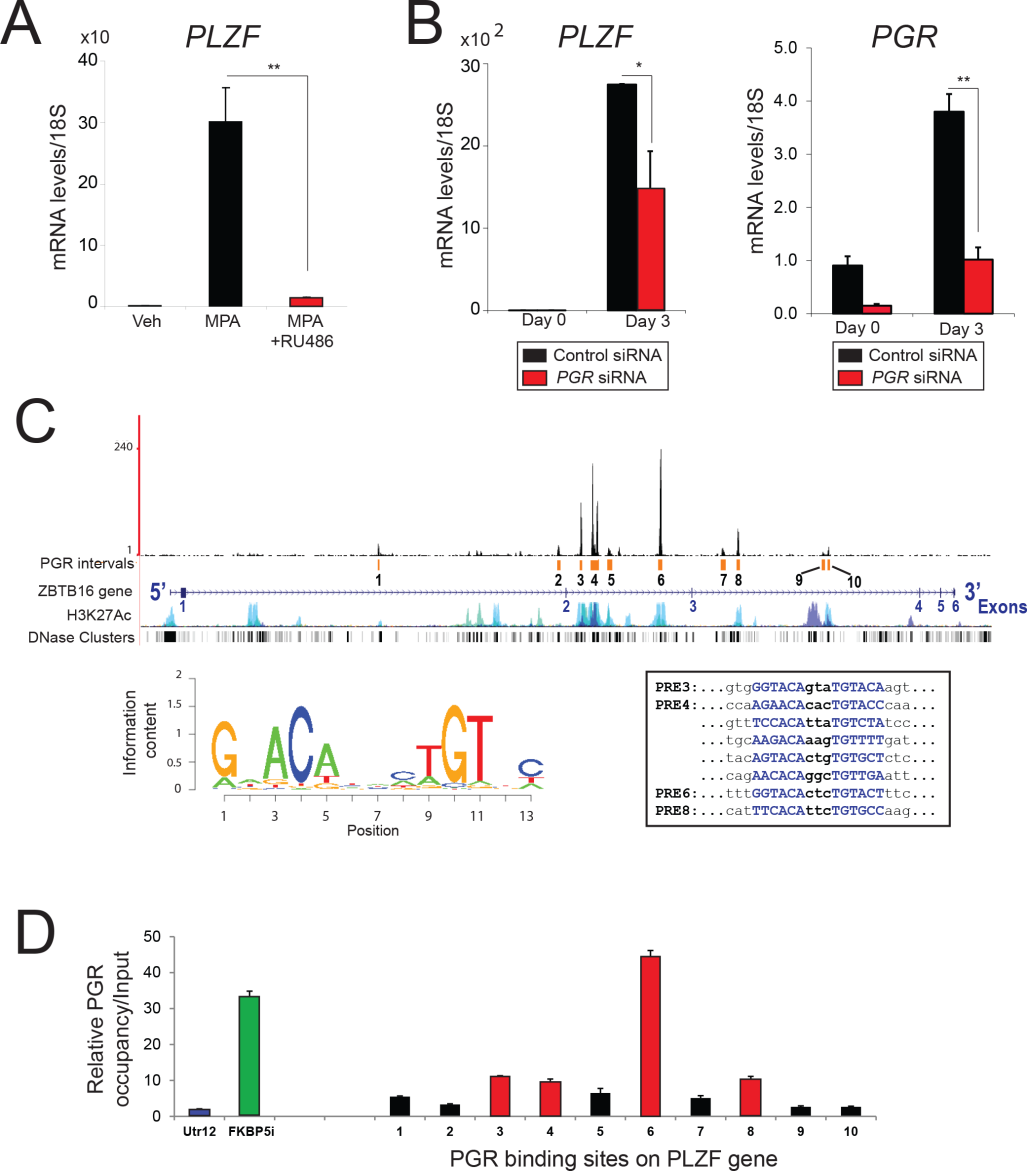
The progesterone receptor mediates progestin induction of *PLZF* expression in hESCs. **A)** Relative transcript levels of *PLZF* in hESCs cultured with vehicle (control), MPA, or MPA in the presence of RU486 for three days in stripped media. Note the marked reduction in MPA-induced *PLZF* transcription when RU486 is included. **B)** *PLZF* and *PR* transcript levels in hESCs transfected with control siRNA or siRNA targeting *PGR* and cultured in EPC media at the indicated time points. Results are reported as the mean ± SE from triplicate samples from one subject (three subjects were tested in total); *P<0.05 and **P<0.01. **C**) Distribution of PGR binding intervals (designated 1 to 10) along the *PLZF* gene locus is shown along with H3K27Ac active sites and DNase hypersensitivity clusters. The sequence logo for progesterone-response element (PRE) is shown along with the sequence of PREs for sites 3, 4, 6, and 8. **D**) Validation of PGR binding to the *PLZF* gene using chromatin immunoprecipitation (ChIP). The histogram shows PCR results following PGR ChIP from hESCs on sites 1 to 10 as depicted in panel C. A non-binding untranslated region (Utr12 (blue bar)) and the FKBP5 intronic region (FKBP5i (green bar)) were used as negative and positive controls respectively for PGR binding.

To confirm that PLZF is rapidly induced by progestin *via* the progesterone receptor (PGR) rather than by rapid non-genomic progestin effects [12], RU486 (a PGR antagonist) and small interfering (si) RNA-mediated knockdown of *PGR* were administered to hESCs (**Fig 2**). Following three days of culture with MPA or MPA plus RU486, *PLZF* transcript levels were examined in hESCs by quantitative real time PCR (**Fig 2A**). Compared to vehicle treatment, MPA significantly increased the transcript levels of *PLZF*; however, this level of induction was markedly attenuated with the co-administration of RU468 (**Fig 2A**). Further confirming the requirement of the PGR in this transcriptional induction, progestin induction of *PLZF* transcription was significantly attenuated with *PGR* knockdown (**Fig 2B**). In addition, acute hormone treatment of the ovariectomized *Pgr* knockout (PRKO) mouse revealed that rapid P4-induced *Plzf* transcription in the murine uterus requires endometrial Pgr (**S2D Fig**). Together, the above results strongly support an indispensable role for PGR in the rapid induction of *PLZF* transcription by progestin or P4 in human and murine uterine cells.

Ten PGR binding sites (labeled 1 to 10 (**Fig 2C**)) in the *PLZF* gene were revealed from an analysis of a recently published chromatin immunoprecipitation-sequencing (ChIP-Seq) dataset derived from PGR cistrome studies on decidualized hESCs [8]. Four of these PGR binding sites (2, 4, 5, and 6) contain consensus progesterone-response elements (PREs) that are enriched in the hESC PGR-cistrome. Chromatin immunoprecipitation assay disclosed significant enrichment in PGR occupancy for sites 3, 4, 6, and 8 within the *PLZF* gene as compared to a negative control region (**Fig 2D**). Collectively, these results suggest that progestin transcriptional induction of *PLZF* involves binding of PGR to multiple sites across the PLZF gene.

### Decidualization of hESCs requires PLZF

**Fig 3:**
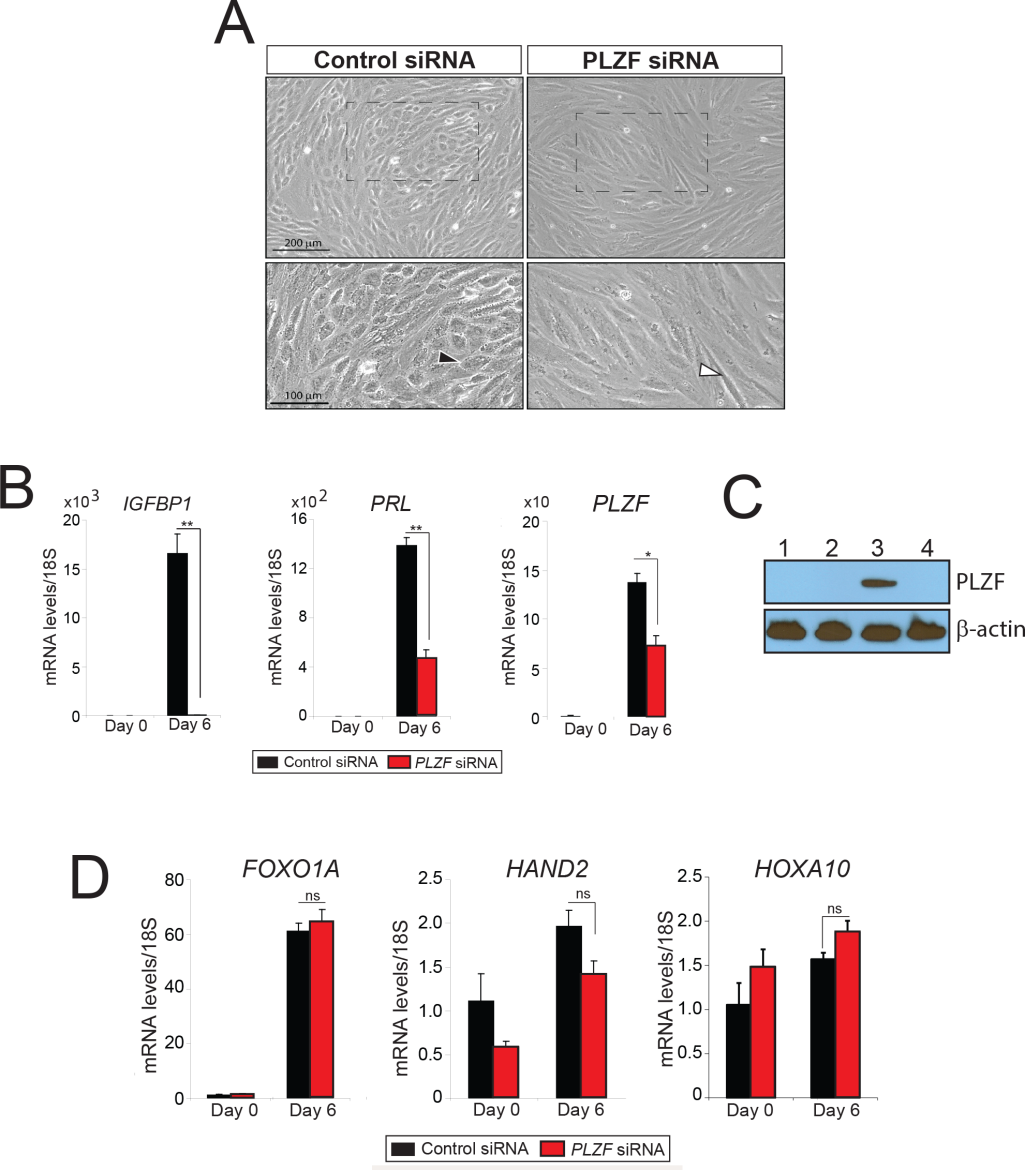
Progestin-dependent decidualization of hESCs requires PLZF. **A)** Cultured for six days in EPC media, the comparative hESC morphology following transfection with control siRNA or *PLZF* siRNA is shown. A typical epithelioid hESC with a polygonal appearance is indicated with a black arrowhead in the presence of control siRNA. A white arrowhead shows a hESC that remains fibroblastic when cultured in the presence of *PLZF* siRNA. Bottom panels represent higher magnifications of regions demarcated by hatched boxes in the corresponding top panels. **B)** Transcript levels of *IGFBP-1*, *PRL*, and *PLZF* in hESCs transfected with control siRNA (black bar) or *PLZF* siRNA (red bar) and cultured in EPC media for the indicated time period. **C)** Western blot analysis of PLZF protein levels to determine the effectiveness of siRNA against PLZF from hESCs transfected with control siRNA or PLZF siRNA and cultured in EPC media. Lanes 1–3 and 2–4 represent hESCs transfected with control siRNA or PLZF siRNA respectively. Lanes 1–2 and 3–4 represent hESCs cultured in EPC media for 0 days and 6 days respectively; β-actin was used as loading control. **D)** Transcript levels of the progesterone-responsive genes: *FOXOA1*, *HAND2*, and *HOXA10* in hESCs transfected with control siRNA or *PLZF* siRNA and cultured in EPC media. Results are reported as the mean ± SE from triplicate samples from one subject (three subjects were tested in total). *P<0.05, **P<0.01, and ns>0.05.

To determine whether PLZF is functionally required for *in vitro* hESC decidualization, siRNA mediated knockdown of *PLZF* transcription was performed in hESCs prior to EPC administration. Compared to control siRNA, hESCs treated with siRNA targeting *PLZF* failed to undergo the typical cellular transformation process in which elongated stromal fibroblasts transform into a polygonal epithelioid morphology, a morphological change which is indicative of stromal decidualization following EPC treatment (**Fig 3A**). Consistent with this block in stromal cell decidualization, a significant attenuation in the induction of decidual biomarkers (*PRL* and *IGFBP-1)* was observed in hESCs with *PLZF* knockdown (**Fig 3B and 3C**). Although these results underscore a critical functional role for PLZF in advancing progestin dependent hESC decidualization, surprisingly the expression level of a number of previously published PGR target genes in hESCs (*FOXO1A*, *HAND2*, and *HOXA10* [13–15]*)* is not changed with *PLZF* knockdown (**Fig 3D**). These results indicate that the PLZF transcription factor is either positioned below these targets in the hierarchy of target genes for PGR action or PLZF operates in parallel to control its own gene target ensemble, which in turn underpins PGR dependent hESC decidualization.

### Identification of the *PLZF* cistrome in hESCs

**Fig 4:**
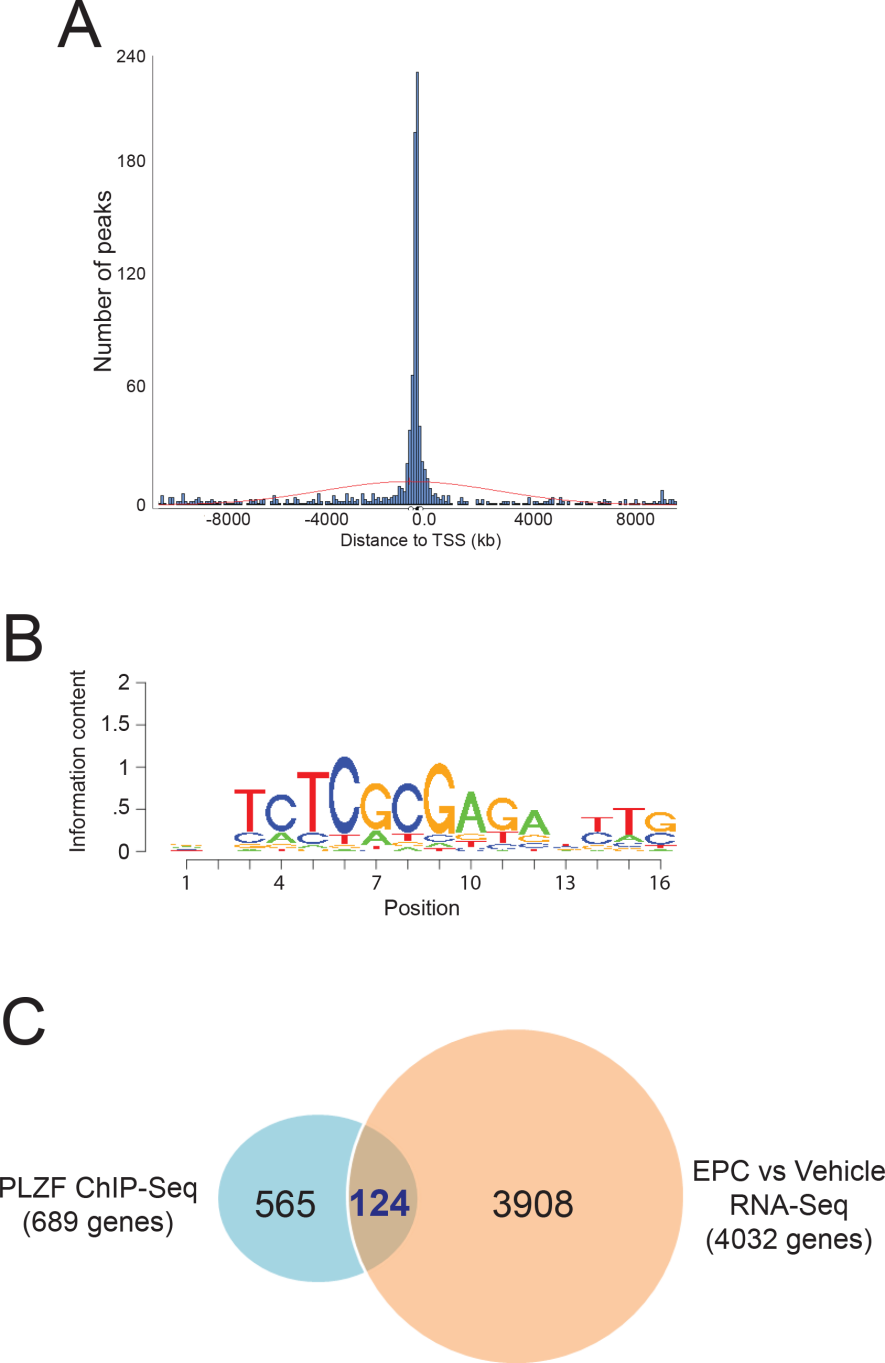
Identification of the PLZF cistrome by ChIP-Seq. **A)** Histogram representing the distance of PLZF binding peaks from the transcriptional start site (TSS) of all genes. **B)** Representative zf-C2H2 DNA binding domain motif (MC00418) identified by SeqPos motif enrichment analysis. **C)** Venn diagram comparing genes bound both by PLZF with genes for which their expression changes in hESCs following EPC induced decidualization [8].

To identify possible PLZF target genes, ChIP-Seq was performed to identify the genome-wide binding events for PLZF in decidualizing hESCs (**Fig 4**). Having passed Illumina’s purity filter, the PLZF cistrome consists of 19,092,785 unique alignments (without duplicate reads) from more than 36 Million reads and aligns with no more than 2 mismatches to the genome. A total of 1,120 sequence intervals are enriched for *PLZF* binding across all chromosomes (supporting information). Peak distribution analyses revealed significant enrichment for PLZF binding near transcription start sites (TSS) with 48% of PLZF intervals located within 500 base pairs (b.p.) of TSS, indicating preferential binding of the PLZF transcription factor to gene promoter regions (**Fig 4A and S1 Table**). DNA sequence motif enrichment analysis revealed the zf-C2H2 DNA binding domain (MC00418) in the top enrichment cluster of motifs in the PLZF cistrome, a common DNA binding motif for many ZBTB transcription factors including PLZF [16] (**Fig 4B**). This cluster also contained DNA binding motifs for breast cancer 1 early onset (BRCA1) (MC00299) and v-ets avian erythroblastosis virus E26 oncogene homolog 1 (ETS1) (MC00018) (**S2 Table**). Following these enrichment groups, are motifs that are commonly found in cistromes of TSS-proximal binding transcription factors, such as motifs recognized by CCAAT-enhancer binding proteins (or C/EBPs), interferon-regulatory factors, SP1, and by leucine zipper family of transcription factors (*i*.*e*. JUN and FOS) (**S2 Table**).

To identify *bona fide* PLZF gene targets, we next compared 689 genes that have at least one PLZF ChIP binding region within 1kb of TSS with genes in hESCs for which expression changed with EPC treatment [8]. From this analysis, we identified 124 genes that are bound by PLZF and are differentially expressed during hESC decidualization (**Fig 4C and S3 Table**). By DAVID and GSEA analysis, the majority of these genes are associated with mitosis and cell cycle progression (**S4 Table**).

### Transcriptional repression of early growth response 1 by PLZF is required for hESC decidualization

**Fig 5:**
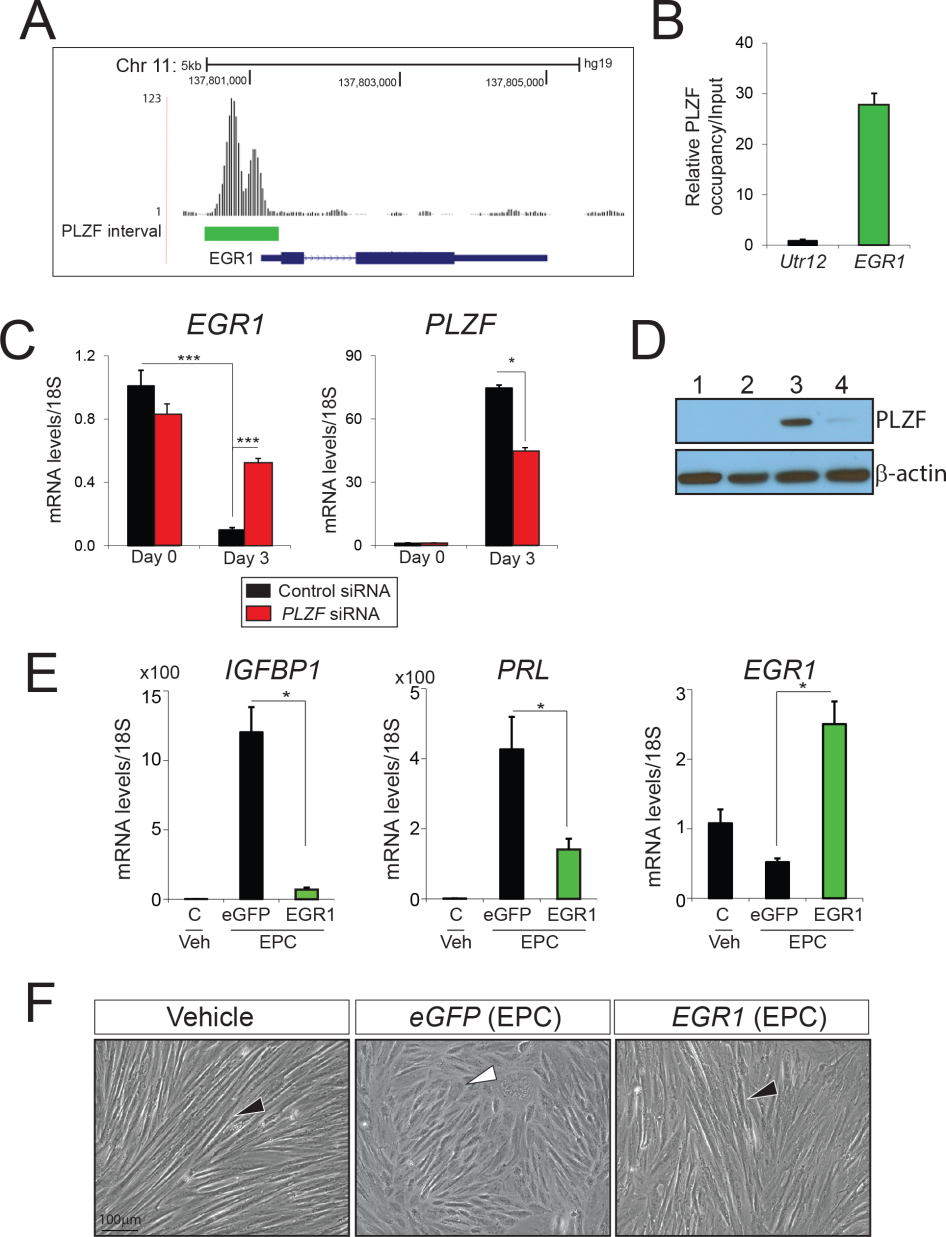
PLZF-mediated transcriptional repression of *EGR1* is critical for hESC decidualization. **A)** Location of PLZF binding sites in the promoter region of *EGR1*. **B)** The histogram shows the qPCR result following ChIP using the PLZF antibody on the *EGR1* promoter region from panel A. **C**) Comparative transcript levels of *EGR1* in hESCs transfected with control siRNA (black bar) or *PLZF* siRNA (red bar) and cultured in EPC media. **D)** Western blot analysis of PLZF protein levels from hESCs transfected with control siRNA (lanes 1–3) or PLZF siRNA (lanes 2–4) cultured in EPC media for 0 days (lanes 1–2) or 3 days (lanes 3–4).; β-actin was used as loading control. **E)** Relative transcript levels of *IGFBP1*, *PRL*, and *EGR1* in non-transduced hESCs or hESCs virally transduced with control eGFP or *EGR1* cDNAs (green bar) and cultured in EPC media. Results are reported as the mean ± SE from triplicate samples from one subject (a total of three subjects were tested). *P<0.05, **P<0.01, and ***P<0.001. **F)** Morphology of hESCs transduced with control eGFP or *EGR1* cDNAs following six days of culture in the presence of the EPC cocktail**;** non-transduced hESCs cultured in vehicle were used as control.

Because PLZF was previously shown to act as a transcriptional repressor of cellular proliferative programs in a number of physiological systems; reviewed in [16], we first focused on genes in the 124 gene list that promote early cellular growth responses and are downregulated during decidualization. The rationale for this prioritization is that a switch from a proliferative to a differentiative phenotype is an essential reprogramming step for hESCs to fully decidualize in response to EPC [7]. Based on the above criteria, we chose Early Growth Response 1 (EGR1) for further study because the gene: (a) ranks in the top five most downregulated gene in the 124 gene list (**S3 Table**); (b) belongs to a small family of transcription factors that enable early cellular growth responses [17]; and (c) exhibits strong PLZF ChIP binding at its promoter (**Fig 5A and 5B**).

In keeping with its role as a driver of cell growth and proliferation, *EGR1* transcript levels are significantly reduced as hESCs differentiate into decidual cells (**Fig 5C**). Interestingly, analysis of our recent RNA-seq data from pre-decidual and decidualized hESCs shows that transcript levels of *EGR1* along with *EGR2* and *EGR3* are significantly reduced with decidualization (**S3A and S3B Fig**); however, only *EGR1* shows PLZF binding (**S3 Table**). Importantly, knockdown of *PLZF* expression results in a significant attenuation in the normal reduction of *EGR1* transcript levels following three days of EPC treatment (**Fig 5C and 5D**), suggesting PLZF represses *EGR1* transcription as hESCs decidualize. Moreover, lentiviral-mediated overexpression of *EGR1* significantly attenuated hESC decidualization in response to EPC treatment as evidenced by a marked reduction in the levels of the decidual biomarkers: *IGFBP1* and *PRL* at the molecular and cellular level (**Fig 5E and 5F**). Interestingly, immunohistochemistry disclosed an inverse correlation between stromal PLZF and EGR1 protein expression in the human endometrium during the E2 dominant proliferative and P4 dominant secretory phase of the menstrual cycle (**S4 Fig**). It should be noted that the human endometrial stroma can decidualize during the secretory phase of the cycle even in the absence of a conceptus [6]. In the late proliferative phase of the cycle, EGR1 expression is detected in both epithelial and stromal cells of the endometrium whereas PLZF expression is not detected (**S4 Fig: compare A-C with G-I**). During the secretory phase of the cycle, significant PLZF expression is found in endometrial stromal cells; however, EGR1 expression is negligible during this time period (**S4 Fig: compare D-F with J-L**). Together, these immunohistochemical findings further support the proposal that PLZF acts as a novel transcriptional repressor of *EGR1* in endometrial stromal cells to enable decidualization.

In the uterus of the ovariectomized mouse, *Egr1* is markedly induced with E2 treatment alone (E2) whereas this induction is significantly blunted with the inclusion of P4 (P4E2) (**S5 Fig**). Further confirming inhibition of E2 induction of *Egr1* by P4, inclusion of RU486 in the P4E2 hormone treatment returns *Egr1* levels to those observed with E2 alone (**S5 Fig**). Interestingly, P4 induction of *PLZF* expression during this period parallels P4 suppression of *Egr1* expression (**S5 Fig**), providing correlative support for the proposal that P4 suppression of E2 induction of uterine *Egr1* is mediated by *PLZF*. Together, our human and mouse studies support the proposal that E2-induced EGR-1 expression is suppressed by P4-induced PLZF in the endometrium.

In sum, recent genome-wide studies at the cistrome and transcriptome level underscore the regulatory complexity of P4-dependent hESC decidualization. Within this complexity, we have uncovered a new signaling pathway (PGR-PLZF-EGR1) that is crucial for P4-driven hESC decidualization.

## Discussion

Extensive transcriptional reprogramming underpins hESC decidualization [8], we report here that induction of PLZF as part of these transcriptional changes is critical for P4-dependent hESC decidualization. Highly conserved in mammals, PLZF is a member of the poxvirus and zinc finger (POZ)/ broad-complex, tramtrack, and bric-`a-brac (BTB) and Kruppel zinc finger (or POK) family of transcription factors; reviewed in [16]. Previous investigations have demonstrated a pleiotropic regulatory role for PLZF in a myriad of developmental programs and physiological responses, from hind-limb skeletal patterning [11] to spermatogonial stem-cell maintenance [18, 19]. Due to its role in limiting cell-cycle progression and cellular proliferation [20, 21], PLZF is known to act as a tumor suppressor in a broad spectrum of malignancies [22–26]. At the transcriptional level, PLZF is known primarily as a repressor; reviewed in [16]; however, an increasing number of studies describe PLZF as an activator of transcription [27–31]. Recruitment of corepressors and subsequent chromatin remodeling has been shown to underlie the repressor function of PLZF [32–34].

In different physiological contexts, PLZF is induced by the steroid hormones: cortisone [35], testosterone [36], and aldosterone [37]. These steroids along with P4 mediate their actions through closely related nuclear receptors belonging to subfamily 3 (Group C) of the nuclear receptor superfamily [38]; note: members of this receptor subfamily bind to a similar DNA response element containing the consensus half-site: AGAACA. Therefore, PLZF most likely evolved as one of a small number of specific target genes for this subclass of steroid hormones, which are known to exert wide-ranging physiological and pathophysiological responses. Although, a previous study reported that glucocorticoids and P4 can induce PLZF transcription in both hESCs and myometrial smooth muscle cells *in vitro* [39], a functional role for PLZF in these uterine cell types was not addressed.

Observed both *in-vitro* and *in-vivo*, endometrial stromal fibroblasts undergo proliferation and differentiation before their transformation into polygonal epithelioid decidual cells [7]. Using an integrative genome-wide approach, we reveal that PLZF is indispensable for P4-dependent hESC decidualization. Indeed, the requirement for PLZF in the morphological and functional transformation of an endometrial stromal fibroblast to an epithelioid decidual cell is reminiscent of its previously reported role in the morphological transformation of embryonic fibroblasts to a more flattened polygonal morphology [40, 41], a change in cell shape which correlates with the acquisition of cellular resistance to oncogenic transformation. Furthermore, the terminal differentiation which results from decidualization is in keeping with PLZF’s role in other physiological systems in which this transcription factor suppresses cell growth and proliferation in favor of differentiation; reviewed in [16].

The rapid P4-induction of PLZF transcription in uterine stromal cells suggests that this transcription factor acts as a direct functional mediator of the P4 signal. This proposal is supported by our ChIP-Seq studies which identified at least ten potential PGR binding sites that span introns 1–3 of the human PLZF gene. This DNA binding pattern concurs with previous studies which show that nuclear receptor binding sites are commonly found in introns located far beyond the confines of the promoter-proximal region of target genes [42, 43]. Looping of the intervening DNA has been posited as one possible mechanism to enable long-range interaction by transcription factors with the promoter-proximal region [44, 45]. Early PLZF induction following progestin/P4 administration also suggests that this transcription factor regulates target gene expression changes that occur early in the P4 signaling cascade. Through the integration of ChIP-Seq and RNA-Seq datasets, we revealed that EGR-1 represents one of these target genes for which its expression level is tightly controlled by PLZF to enable normal hESC decidualization by P4 exposure.

Recognizing the GC-rich consensus sequence GCG(T/G)GGGCG in promoter regions of target genes, EGR1 (also known as NGFI-A, Zif 268, or Krox 24) is a Cys_2_-His_2_ zinc finger transcription factor, which belongs to an immediate early response gene family that includes EGR 2–4 [46]. A wide spectrum of extracellular signaling cues activates EGR1, which in turn modulates cellular proliferation, differentiation, and apoptosis in diverse target tissues [17]. For example, EGR1 is induced by E2 in MCF-7 cells [47], suggesting that EGR1 is a mediator of the established E2 mitogenic effects in this human breast cancer cell line. Studies on the Egr1 knockout mouse reveal that Egr1 is required for normal follicular development, ovulation, and luteinization [48]; however, uterine functionality was not assessed. Despite the aforementioned, earlier studies demonstrated that Egr1 is rapidly induced in the rat uterus by E2 [49, 50]_,_ suggesting that Egr1 may mediate the known mitogenic effects of E2 in this tissue. Interestingly, results from these studies are similar to our findings in the mouse in which Egr1 is rapidly induced to high levels by E2 in the uterus; however, we further showed that this induction is also markedly reduced by P4 with simultaneous induction of PLZF. Intriguingly, recent investigations show that unscheduled elevation of EGR1 levels compromises hESC viability *in vitro* [51], indicating that strict controls on the levels of this transcription factor are critical for normal hESC function. These data may explain the need to reduce the expression levels of EGR1 as the pre-decidual fibroblast transforms into a decidual cell and underscores the importance of the P4:PGR: PLZF signaling axis in this regulatory process. Recent mouse studies further support the above by showing that while Egr1 is strongly expressed in the subluminal stroma surrounding the implanting blastocyst during early pregnancy [52], Egr1 expression is significantly reduced following *in vivo* and *in vitro* artificial decidualization [52]. Moreover, Egr1 overexpression downregulates the expression of the decidual marker decidual/trophoblast prolactin-related protein (Dtprp) in murine uterine stromal cells, while inhibition of Egr1 upregulated the expression of Dtprp under *in vitro* decidualization conditions [52]. Collectively, these results agree with our findings in hESCs in which overexpression of Egr1 blocks decidualization and significantly attenuates the induction of the decidual biomarkers, PRL and IGFBP-1.

Interestingly, we demonstrate that PLZF knockdown does not change the expression levels of FOXO1A, HOXA10 or HAND2 during hESC decidualization; these three genes have been shown to be important for the hESC decidual response [14, 53–57]. We also reveal that knockdown of FOXO1A, HAND2 or HOXA10 expression levels does not affect PLZF induction during hESC decidualization, suggesting that PLZF operates in a separate parallel pathway to FOXO1A, HAND2 and HOXA10 signaling. As further support for this conclusion, previous global transcript profiling of hESCs following FOXO1A or HOXA10 knockdown did not show changes in PLZF expression levels [58, 59]. In related studies, our ChIP-Seq analysis did not detect PLZF binding sites within the IGFBP1 or PRL genes, indicating that the downregulation of IGFBP1 and PRL expression following PLZF knockdown is through an indirect signaling axis and is not through FOXOA1 regulation of these decidual biomarkers [60].

In conclusion, pregnancy success relies on the execution of a programmed sequence of events, with uterine decidualization following embryo implantation representing a crucial early event which ensures not only the establishment but also the long-term maintenance of the fetomaternal interface. Advancement in our mechanistic understanding of P4’s involvement in decidualization is predicated upon identifying the key effectors that mediate the P4 signal into a decidual response. An integrative analysis of genome-scale data, together with primary hESC studies, we disclose PLZF as a potent mediator of P4 responsiveness which is essential for hESC decidualization. We also reveal that PLZF controls EGR-1 expression levels, the perturbation of which can compromise decidual progression. Given that further understanding of the molecular mechanisms that underlie P4-driven decidualization is essential if we are to formulate new clinical modalities in the diagnosis, prognosis, and treatment of infertility based on a defective endometrium, we believe our findings offer a new mechanistic perspective on P4 responsiveness in the uterus which may contribute to novel fertility solutions in the future.

## Materials and Methods

### Human endometrial stromal cell isolation

For *in vitro* decidualization studies, endometrial biopsies were obtained using a pipelle catheter or curette under sterile conditions from the uterine fundus of healthy women of reproductive age during the proliferative phase of their menstrual cycle. Subjects ranged in age between 27–38 years and had a normal uterus as evaluated by transvaginal ultrasound. Volunteers provided written informed consent prior to endometrial tissue biopsy, which was conducted in accordance with a protocol approved by the Institutional Review Board (IRB) at Baylor College of Medicine and the guidelines of the Declaration of Helsinki [61]. A portion of the endometrial biopsy was used for immunohistochemistry (see below). The remainder of the biopsy was used to prepare human endometrial stromal cells (hESCs) as previously described [9, 62]. Isolated hESCs were cultured in DMEM/F-12 media containing 10% fetal bovine serum (FBS), 100 units/ml penicillin, and 0.1 mg/ml streptomycin (termed hESC media); studies were conducted with early passage primary hESCs (≤ four passages). For each experiment, at least three individual subjects provided the endometrial biopsy samples for cell preparation. For immunohistochemical analysis, endometrial biopsies also were taken from volunteers during the mid-secretory phase of their cycle (day 19–23) and fixed in formalin overnight (along with proliferative phase endometrial tissue) before paraffin embedding the following day.

### Decidualization of hESCs and siRNA transfection

For RNA interference based studies, hESCs were transfected in triplicate with non-targeting (or control) small interfering (si)RNAs (D-001810-10-05), siRNAs targeting *PLZF* (L-005196-00-0005), or *PGR* (L-006763-00-0005) or *FOXO1A* (L-003006-00-0005) or *HAND2* (L-008698-02-0005) or *HOXA10* (L-006336-00-0005) (Thermo Scientific, Dharmacon RNAi Technologies, Chicago, IL) in six-well culture plates. In each well, siRNAs (60 pmol) were transfected into hESCs using Lipofectamine 2000 reagent in 1× Opti-MEM I reduced-serum media (Invitrogen Corporation). Forty-eight hours after transfection, *in vitro* decidualization of hESCs was initiated by treating cells with 1× Opti-MEM I reduced-serum media containing 2% FBS, 10nM E2, 1μM MPA (Sigma-Aldrich), and 50 μM cAMP (Sigma-Aldrich) (termed EPC media). The EPC medium was changed every two days, and hESCs harvested at defined time points as indicated [62]. Quantitation of *PRL* and *IGFBP-1* transcript levels served as a positive molecular readout for hESC decidualization.

### ChIP-seq data analyses and validation

Human ESCs were cultured in EPC media for 72 hours before PLZF and Input ChIP-Seq were performed by Active Motif, Inc. (Carlsbad, CA). Human ESCs derived from six subjects were pooled before being fixed in 4% formaldehyde, snap-frozen, and shipped on dry ice to Active Motif. A goat polyclonal anti-human PLZF antibody (sc-11146; Santa Cruz Biotechnology, Inc.) was used for the PLZF ChIP assay. Enriched DNA from the ChIP assay was amplified and DNA libraries were sequenced on an Illumina platform, followed by alignment of sequenced reads (24 million reads) to the human genome (GRCh37/hg19, February 2009). Peak calling was performed by applying a threshold of 18 (5 consecutive bins containing 0.18 aligns) before the resultant-called peaks were stored as Browser Extendable Data (BED) files. Due to the pronounced enrichment for TSS-proximal binding of PLZF by Cis-regulatory Element Annotation System (CEAS) analysis, genes associated with PLZF binding were called if the ChIP binding intervals were located within 950 bp from the TSS. Galaxy/Cistrome’s implementation of the Cis-regulatory Element Annotation System (CEAS) and Genomic Regions Enrichment of Annotations Tool (GREAT) tools were used for enrichment analyses on chromosomes and genomic annotations. Database for Annotation, Visualization, and Integrated Discovery Bioinformatics Resources (DAVID) v6.7 (http://david.abcc.ncifcrf.gov/)) and Gene Set Enrichment Analysis (GSEA) on Molecular Signatures Databases (MSigDB) collection (http://software.broadinstitute.org/gsea/index.jsp) analyses were performed on protein coding genes to identify enriched biological themes. Integration of PLZF ChIP-Seq data with RNA-Seq data sets was performed using a relational database system [63]. ChIP validation assays were performed on hESCs cultured in EPC media for seventy-two hours (Active Motif). A goat polyclonal anti-human PLZF antibody and a rabbit anti-human PGR (H-190) antibody (sc-7208, Santa Cruz Biotechnology, Inc.) were used to immunoprecipitate PLZF and PR proteins with associated bound DNA from fragmented chromatin respectively. Enriched DNA fragments from the PLZF and PGR ChIP assays were subjected to standard PCR analyses using specific primers (S5 Table) for specific binding regions. Reverse cross-linked chromatin fragments that were not immunoprecipitated (input DNA) were used as an internal control.

### Molecular analyses

For quantitative real time PCR studies, total RNA was isolated from cells or tissue using the RNeasy total RNA isolation kit (Qiagen Inc., Valencia, CA). Complementary DNA (cDNA) was synthesized using the Bio-Rad Reverse Transcription kit (Bio-Rad Laboratories, Inc., Hercules, CA). Quantitative real time PCR analyses were performed using validated primers (Applied Biosystems/Life Technologies, Grand Island, NY) and TaqMan 2× master mix; ribosomal RNA (18S) was used as an internal control for gene specific primers; the primer list is shown in S6 Table.

For western analyses, total protein was extracted from uterine tissue using 1× protein lysis buffer (0.5% sodium deoxycholate, 1% Nonidet P-40, 0.1% SDS, phosphate-buffered saline (PBS), pH 7.4). An equal amount of protein extract (20 μg) was run on 4–15% gradient SDS-PAGE gels (Bio-Rad Laboratories, Inc.) and transferred to polyvinylidene difluoride (PVDF) membranes. Membranes were blocked with 1× TBS containing 0.5% Tween-20 and 5% non-fat dry milk powder. Subsequently, membranes were incubated with rabbit polyclonal anti-PLZF (Santa Cruz Biotechnology, Inc.) or rabbit anti-human PGR (H-190) antibody (Santa Cruz Biotechnology, Inc.) or monoclonal anti-β-actin (Sigma-Aldrich) antibodies to detect PLZF, PGR and β-actin proteins respectively. Horseradish peroxidase-conjugated antibodies (Santa Cruz Biotechnology, Inc.) were used as secondary antibodies and the SuperSignal West Pico Chemiluminescent Substrate kit (Pierce Biotechnology, Rockford, IL) was used to detect the chemiluminescent signal.

### Immunofluorescence cytology

When hESCs reached 60–70% confluence on eight-well chamber slides in triplicate wells, media was changed to EPC media. The EPC media was changed every two days for a total of six days before hESCs were fixed in 2% paraformaldehyde and permeabilized in 0.2% Triton-X detergent. Cells were washed and blocked in 1× PBS with 0.5% normal goat serum before incubation overnight with the rabbit polyclonal anti-human PLZF primary antibody ((H-300) Santa Cruz Biotechnology, Inc.). To detect immunoreactivity, hESCs were incubated with the Alexa Fluor 488-conjugated goat anti-rabbit secondary antibody (A-11008; Life Technologies) to visualize immunopositive cells (green fluorescence). Cell nuclei were visualized using the 4, 6-diamidino-2-phenylindole stain (DAPI; Sigma-Aldrich). Finally, slides were separated from the chambers and mounted using Slowfade mounting media before imaging.

### Lentivirus transduction

A lentiviral vector expressing EGR1 (EX-OL00487-LX304) and a control vector expressing EGFP (EX-EGFP-LX304-B) were purchased from GeneCopoeia (Rockville, MD). Lentivirus was produced in the Gene Vector Core at Baylor College of Medicine. Twenty four hours following transduction of hESCs with control virus harboring empty vector or lentivirus expressing EGR1, media was replaced with EPC media to induce decidualization; lentiviral-mediated EGR1 overexpression was confirmed by real-time PCR.

### Histological analyses

Briefly, paraformaldehyde-fixed tissues were processed and embedded in paraffin as previously described [62]. Tissue blocks were serially sectioned into 5-μm thick sections that were placed on Superfrost Plus glass slides (Fisher Scientific, Inc., Pittsburgh, PA). For immunohistochemical analyses, sections were deparaffinized before being rehydrated and boiled in a citric acid-based antigen unmasking solution (Vector Laboratories, Inc., Burlingame, CA). After blocking, sections were incubated overnight at 4°C with a rabbit polyclonal anti-human PLZF (H-300) antibody (1:150 dilution; sc-22839, Santa Cruz Biotechnology Inc., Dallas, TX) or anti-human EGR1 (#15F7) antibody (1:150 dilution; Cell Signaling, Danvers, MA). After incubation with the primary antibodies, sections were incubated with the goat anti-rabbit IgG secondary antibody (Vector Laboratories, Inc.), followed by incubation with ZyMax streptavidin-horseradish peroxidase conjugate (Invitrogen Corporation, Carlsbad, CA). The 3, 3’-diaminobenzidine (DAB) peroxidase substrate kit (Vector Laboratories, Inc.) was used to visualize immunoreactivity before sections were counterstained with hematoxylin. Cover slips were mounted onto stained sections using Slowfade mounting media (Fisher Scientific, Inc.).

### Mice and hormone treatments

In a recurrent photocycle of 12h lights on-off, mice were housed in temperature controlled (22°C ± 2°C) rooms in an AAALAC accredited *vivarium* maintained by the Center for Comparative Medicine at the Baylor College of Medicine. A diet of irradiated Tekland global soy protein-free extruded rodent food pellets (Harlan Laboratories Inc., Indianapolis, IN) and fresh water were provided *ad libitum* to mice. Animal studies were undertaken following the guidelines described in the Guide for the Care and Use of Laboratory Animals (published by the National Research Council (Eighth Edition 2011)). Animal protocols (AN-1513; AN-544; and AN-4203) used in this research received prior approval from the Institutional Animal Care and Use Committee (IACUC) at Baylor College of Medicine. The PRKO mouse has been previously described [64]. The following anesthesia was used for surgery: ketamine 37.5mg, xylazine 1.9mg, and acepromazine 0.37 mg sq to 5ml with 2.45ml sterile water given at 0.75–1.5ml/kg BW, IP. Mice were euthanized by cervical disarticulation while under the plane of anesthesia; CO_2_ euthanasia was achieved with automated CO_2_ euthanasia chambers (EUTHANEX).

To evaluate uterine responsiveness to steroid hormone, mice were ovariectomized at six weeks-of-age and then rested for two weeks to ensure removal of serum ovarian steroid hormones before administration of exogenous hormone. Subcutaneous (s.c.) injection within the interscapular region of 100 μl sesame oil (vehicle control), 1 mg P4 (Sigma-Aldrich, St. Louis, MO), 100 ng E2 (Sigma), or 1mg RU486 (Sigma) in 100 μl vehicle was used to administer hormones and the antiprogestin. Following hormone treatment, whole uterine tissue was processed from euthanized mice for RNA, protein, and/or histological analysis.

### Statistical analyses

For statistical analyses, a two-tailed student’s t test and one-way ANOVA with Tukey’s *post-hoc* test were performed using the Instat tool package version 3.0 (GraphPad software Inc., La Jolla, CA). Data with p values less than 0.05 were considered statistically significant.

## Supporting Information

S1 Fig Rapid induction of PLZF by progestin in hESCs. **A)** Table shows the change in *ZBTB16* (*PLZF*), *IGFBP1*, and *PRL* expression in hESCs during decidualization from a recently published RNA-seq dataset [8]. The relative gene expression change in hESCs following treatment with the decidual stimulus (EPC) compared to vehicle treatment is represented as a Log2 transformed fold change value. **B)** Relative transcript levels of *PLZF* in hESCs treated with MPA alone at indicated time points. **C)** Relative transcript levels of *PLZF* in hESCs pre-treated with cyclohexamide (10ug/ML) for an hour and treated with MPA for four hours as indicated. **D)** Comparative protein levels of PGR in hESCs cultured for zero days (lanes 1 and 2) or four days (lanes 3 and 4) or six days (lanes 5 and 6) with the decidual stimulus (EPC; lanes 3 and 5) or MPA and cAMP (PC; lanes 4 and 6) compared to vehicle treatment cells (lanes 1 and 2). Note: The PGR isoforms (A and B) are elevated in EPC and PC treated cells; β-actin was used as loading control.
(TIF)

S2 Fig Progesterone induction of *Plzf* in the murine endometrium requires the progesterone receptor. **A)** Relative *Plzf* transcript levels in uteri from ovariectomized wild-type mice treated with sesame oil (vehicle control) or progesterone for the indicated times. **B)** Western blot analyses for Plzf and β-actin protein levels in uteri from ovariectomized wild-type mice treated with sesame oil (vehicle control) or progesterone for the indicated times. Note: uteri from ovariectomized PRKO mice treated with progesterone for 6 hours were used as Pgr negative controls. **C)** Immunohistochemical detection of Plzf in uteri from adult virgin and early pregnant (5 days post coitum (d.p.c)) mice. Note the low levels of Plzf expression in the uterus of the virgin mouse compared to striking expression levels of Plzf in the stromal compartment of uterus of the early pregnant mouse (white arrow). At this stage of pregnancy, however, expression of Plzf was not detected in the luminal or glandular epithelium (LE and GE respectively); scale bar denotes 100μm and applies to all panels. **D)** Relative *Plzf* transcript levels in uteri from ovariectomized wild type control and PRKO mice injected with sesame oil (vehicle control) or progesterone and euthanized 6 hours post-injection. Results represent the mean ± SE; n = 3 mice/group. *P<0.05, **P<0.01, ns = not significant.
(TIF)

S3 Fig **A)** Table depicting *EGR1*, *EGR2*, and *EGR3* transcript expression changes during EPC-driven decidualization [8]; gene expression changes are represented as Log2 transformed fold change. **B)** Relative transcript levels of *EGR1*, *EGR2*, *EGR3*, and *EGR4* in hESCs cultured for 3 days in vehicle control (red bar) or EPC cocktail (black bar).
(TIF)

S4 Fig An inverse expression profile for PLZF and EGR1 in the human endometrium during the proliferative and secretory phase of the menstrual cycle. Immunohistochemical detection of PLZF (A to F) and EGR1 (G to L) expression in human endometrial tissue biopsied during proliferative phase (A-C and G-I) and secretory phase (D-F and J-I) of the menstrual cycle. Black and white arrowheads indicate glandular epithelium and stroma respectively; scale bar in panel A indicates 100 μm and applies to all panels.
(TIF)

S5 Fig Progesterone induction of *Plzf* is associated with the inhibition of estrogen induced Egr1 expression in the murine endometrium. Relative *Plzf* and *Egr1* transcript levels in uteri from ovariectomized wild-type mice treated with sesame oil (vehicle control) or E2 (3 h) or P4E2 in presence or absence of RU486. Note: The RU486 antagonist was added 30 min prior to P4 treatment (P4 was administered 3 hours prior to E2 treatment). Results represent the mean ± SE; n = 3 mice/group. *P<0.05, **P<0.01, ns = not significant.
(TIF)

S6 Fig Expression of PLZF is not controlled by FOXOA1, HAND2, and HOXA10 in hESCs. **A)** Transcript levels of *PLZF* in hESCs transfected with control siRNA or *FOXO1A* siRNA, *HAND2 siRNA*, or *HOXA10 siRNA* and cultured in EPC media for the indicated time period. **B)** Efficient knockdown of FOXOA1, HAND2, and HOXA10 was confirmed with the measurement of transcript levels of *FOXOA1*, *HAND2*, and *HOXA10* in hESCs transfected with siRNA against respective protein as indicated. Results are reported as the mean ± SE from triplicates. *P<0.05, **P<0.01and ns>0.05.
(TIF)

S1 Table Genome-wide binding of PLZF represented as log2 ratio to the expected genomic distribution using Cis-regulatory Element Annotation System (CEAS) analysis (http://cistrome.org/ap/). (XLS)

S2 Table SeqPos Output for Motifs enriched in PLZF Intervals. (XLS)

S3 Table List of PLZF bound genes within 1kb of the TSS that were significantly changed with EPC treatment in hESCs. (XLS)

S4 Table DAVID Gene Ontology and GSEA analysis of PLZF bound genes within 1kb of the TSS. (XLS)

S5 Table List of primers used for ChIP qPCR validations. (DOC)

S6 Table Primer list. (DOC)

S1 Dataset BED format files which consists of genomic position information of input and PLZF ChIP intervals. (ZIP)
