# Supplementary figures and images for: The Promyelocytic Leukemia Zinc Finger Transcription Factor Is Critical for Human Endometrial Stromal Cell Decidualization
Source: PLoS Genet. 2016 Apr 1;12(4):e1005937. doi: 10.1371/journal.pgen.1005937 (PMC4817989; doi:10.1371/journal.pgen.1005937)

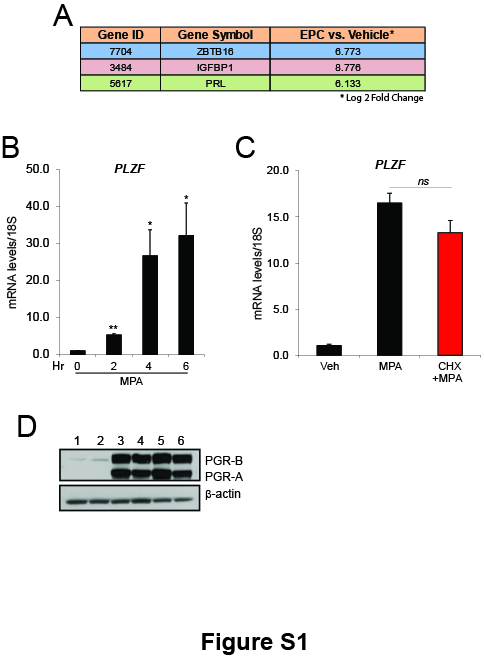

Supplement: S1 Fig — A) Table shows the change in ZBTB16 (PLZF), IGFBP1, and PRL expression in hESCs during decidualization from a recently published RNA-seq dataset [8]. The relative gene expression change in hESCs following treatment with the decidual stimulus (EPC) compared to vehicle treatment is represented as a Log2 transformed fold change value. B) Relative transcript levels of PLZF in hESCs treated with MPA alone at indicated time points. C) Relative transcript levels of PLZF in hESCs pre-treated with cyclohexamide (10ug/ML) for an hour and treated with MPA for four hours as indicated. D) Comparative protein levels of PGR in hESCs cultured for zero days (lanes 1 and 2) or four days (lanes 3 and 4) or six days (lanes 5 and 6) with the decidual stimulus (EPC; lanes 3 and 5) or MPA and cAMP (PC; lanes 4 and 6) compared to vehicle treatment cells (lanes 1 and 2). Note: The PGR isoforms (A and B) are elevated in EPC and PC treated cells; β-actin was used as loading control. (TIF) [file pgen.1005937.s001.tif]

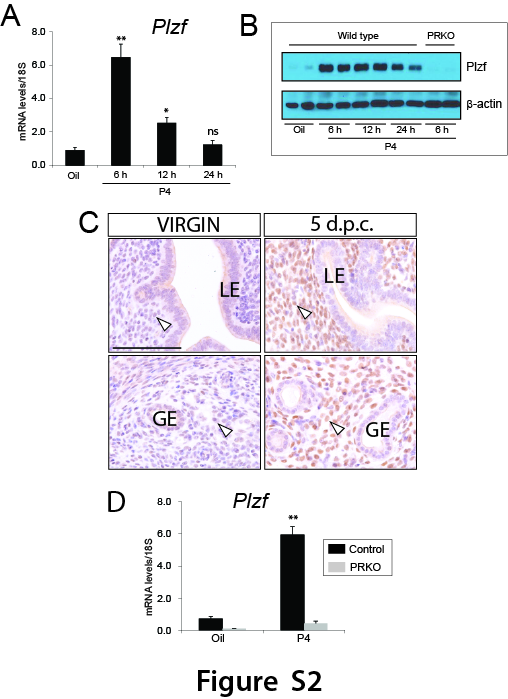

Supplement: S2 Fig — A) Relative Plzf transcript levels in uteri from ovariectomized wild-type mice treated with sesame oil (vehicle control) or progesterone for the indicated times. B) Western blot analyses for Plzf and β-actin protein levels in uteri from ovariectomized wild-type mice treated with sesame oil (vehicle control) or progesterone for the indicated times. Note: uteri from ovariectomized PRKO mice treated with progesterone for 6 hours were used as Pgr negative controls. C) Immunohistochemical detection of Plzf in uteri from adult virgin and early pregnant (5 days post coitum (d.p.c)) mice. Note the low levels of Plzf expression in the uterus of the virgin mouse compared to striking expression levels of Plzf in the stromal compartment of uterus of the early pregnant mouse (white arrow). At this stage of pregnancy, however, expression of Plzf was not detected in the luminal or glandular epithelium (LE and GE respectively); scale bar denotes 100μm and applies to all panels. D) Relative Plzf transcript levels in uteri from ovariectomized wild type control and PRKO mice injected with sesame oil (vehicle control) or progesterone and euthanized 6 hours post-injection. Results represent the mean ± SE; n = 3 mice/group. *P<0.05, **P<0.01, ns = not significant. (TIF) [file pgen.1005937.s002.tif]

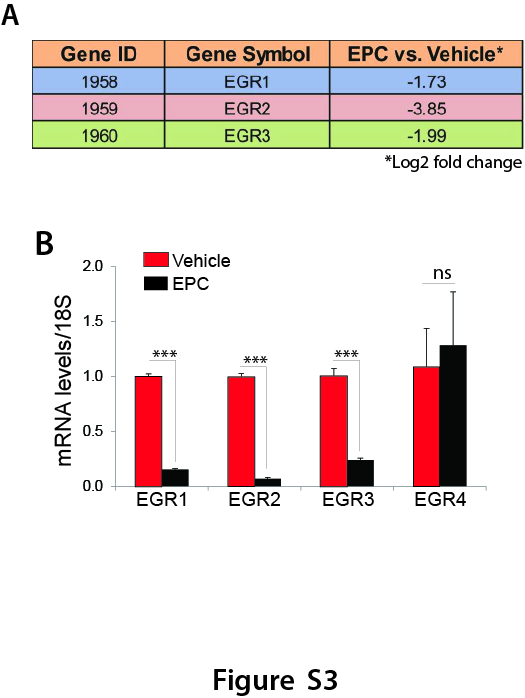

Supplement: S3 Fig — A) Table depicting EGR1, EGR2, and EGR3 transcript expression changes during EPC-driven decidualization [8]; gene expression changes are represented as Log2 transformed fold change. B) Relative transcript levels of EGR1, EGR2, EGR3, and EGR4 in hESCs cultured for 3 days in vehicle control (red bar) or EPC cocktail (black bar). (TIF) [file pgen.1005937.s003.tif]

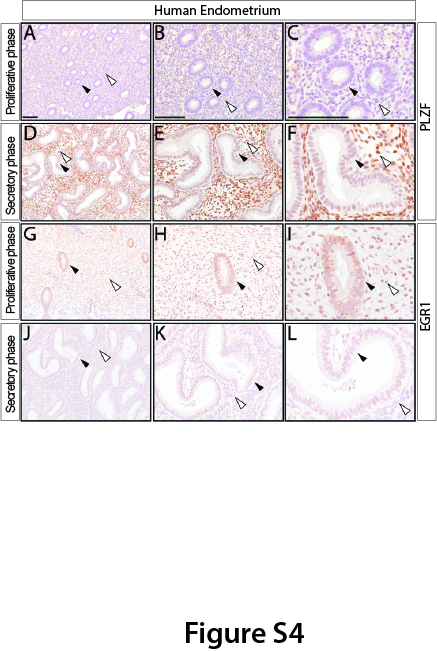

Supplement: S4 Fig — Immunohistochemical detection of PLZF (A to F) and EGR1 (G to L) expression in human endometrial tissue biopsied during proliferative phase (A-C and G-I) and secretory phase (D-F and J-I) of the menstrual cycle. Black and white arrowheads indicate glandular epithelium and stroma respectively; scale bar in panel A indicates 100 μm and applies to all panels. (TIF) [file pgen.1005937.s004.tif]

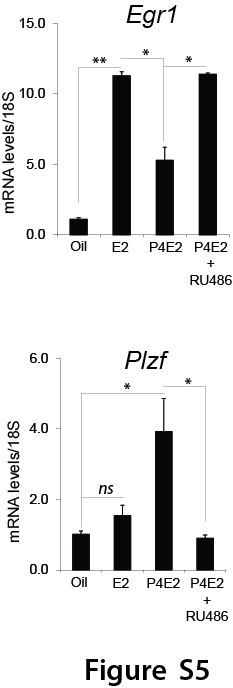

Supplement: S5 Fig — Relative Plzf and Egr1 transcript levels in uteri from ovariectomized wild-type mice treated with sesame oil (vehicle control) or E2 (3 h) or P4E2 in presence or absence of RU486. Note: The RU486 antagonist was added 30 min prior to P4 treatment (P4 was administered 3 hours prior to E2 treatment). Results represent the mean ± SE; n = 3 mice/group. *P<0.05, **P<0.01, ns = not significant. (TIF) [file pgen.1005937.s005.tif]

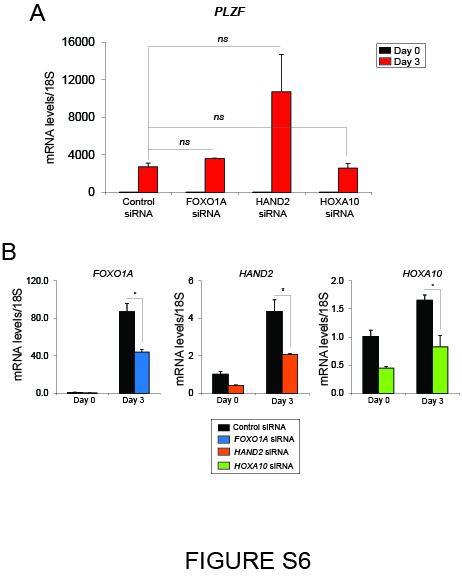

Supplement: S6 Fig — A) Transcript levels of PLZF in hESCs transfected with control siRNA or FOXO1A siRNA, HAND2 siRNA, or HOXA10 siRNA and cultured in EPC media for the indicated time period. B) Efficient knockdown of FOXOA1, HAND2, and HOXA10 was confirmed with the measurement of transcript levels of FOXOA1, HAND2, and HOXA10 in hESCs transfected with siRNA against respective protein as indicated. Results are reported as the mean ± SE from triplicates. *P<0.05, **P<0.01and ns>0.05. (TIF) [file pgen.1005937.s006.tif]
