# Supplementary material for: The Promyelocytic Leukemia Zinc Finger Transcription Factor Is Critical for Human Endometrial Stromal Cell Decidualization
Source: PLoS Genet. 2016 Apr 1;12(4):e1005937. doi: 10.1371/journal.pgen.1005937 (PMC4817989; doi:10.1371/journal.pgen.1005937)
Supplement: S5 Table — (DOC) [file pgen.1005937.s011.doc]

| **Site** | **Primer Sequence** |
| --- | --- |
|  |  |
| ZBTB16 PR site 1 | AAGGAAGGTCACAAAACATGAG |
|  | ACATCACGTCCCCAAGAAAC |
|  |  |
| ZBTB16 PR site 2 | CTATCAGAAGGCCGTACACAG |
|  | GCCTCCCTAGAGCCACAGTC |
|  |  |
| ZBTB16 PR site 3 | TTGAGAATAGGCCGCATTGA |
|  | GGAAAAACTGCGAAGGAAGA |
|  |  |
| ZBTB16 PR site 4 | TCCACACAAACCCTGTCTCA |
|  | CCTCTAGGGCACCATTCATT |
|  |  |
| ZBTB16 PR site 5 | CCTCTAGGGCACCATTCATT |
|  | GTCTGAGCCCCAAAGGTCAT |
|  |  |
| ZBTB16 PR site 6 | CCTTGAGGGAAAGAACACAC |
|  | TCAGACGGAGAACAGCACAC |
|  |  |
| ZBTB16 PR site 7 | TGGATTTCTGTCCTGGTGTG |
|  | TAAGCGTGGCCTCCTAGAAC |
|  |  |
| ZBTB16 PR site 8 | GGGAGGACAGGACAGTGAGT |
|  | GTTTTCAATGGGGAGGTCAC |
|  |  |
| ZBTB16 PR site 9 | TGTTGCCTGTTGCCATAAAC |
|  | GCTCTCACTTTACGCCTTGTG |
|  |  |
| ZBTB16 PR site 10 | GACGGAGGGGTCGAGTTAAG |
|  | TGATTTATTCCAAGGACCATCTG |
|  |  |
| FKBP5 | Caaatccccacaaagtcaaac |
|  | gttttgcagctcgcttgatg |
|  |  |
| EGR1 PLZF site | GCAGCACCTTATTTGGAGTG |
|  | CTTCTTCCCTCCTCCCAGAG |
|  |  |
| Untr12 | Active Motif Catalog number 71001 |
