# Supplementary material for: The Promyelocytic Leukemia Zinc Finger Transcription Factor Is Critical for Human Endometrial Stromal Cell Decidualization
Source: PLoS Genet. 2016 Apr 1;12(4):e1005937. doi: 10.1371/journal.pgen.1005937 (PMC4817989; doi:10.1371/journal.pgen.1005937)
Supplement: S6 Table — (DOC) [file pgen.1005937.s012.doc]

| **Gene** | **Catalog number** |
| --- | --- |
| *IGFBP1* | Hs00426285_m1 |
| *PRL* | Hs00168730_m1 |
| *PLZF* | Hs00957433_m1 |
| *EGR1* | Hs00152928_m1 |
| *FOXO1A* | Hs01054576_m1 |
| *HAND2* | Hs00232769_m1 |
| *HOXA10* | Hs00172012_m1 |
| *PGR* | Hs01556702_m1 |
| *Plzf* | Mm01176868_m1 |
| *Egr1* | Mm00656724_m1 |
